# Tissue oxygen saturation changes and postoperative complications in cardiac surgery: a prospective observational study

**DOI:** 10.1186/s12871-019-0905-5

**Published:** 2019-12-16

**Authors:** Sabino Scolletta, Federico Franchi, Elisa Damiani, Armando Cennamo, Roberta Domizi, Antonio Meola, Claudia Scorcella, Davide Vanoli, Christopher Münch, Erica Adrario, Luca Marchetti, Fabio Silvio Taccone, Abele Donati

**Affiliations:** 1 Department of Medicine, Surgery and Neuroscience, Anesthesia and Intensive Care Unit, University of Siena, Via Bracci 1, 53100 Siena, Italy; 2 Department of Biomedical Sciences and Public Health, Clinic of Anesthesiology and Intensive Care, AOU Ospedali Riuniti di Ancona, Università Politecnica delle Marche, via Conca 71, 60126 Torrette di Ancona, Ancona, Italy; 3 Cardiac Anesthesia and Intensive Care Unit, AOU Ospedali Riuniti di Ancona, via Tronto 10/a, 60126 Torrette di Ancona, Ancona, Italy; 4 Department of Intensive Care, Université Libre de Bruxelles, Hospital Erasme, Route de Lennik, 808 –, 1070 Brussels, Belgium

**Keywords:** Cardiac surgery, Tissue oxygenation, Near InfraRed spectroscopy, Postoperative complications

## Abstract

**Background:**

Cardiac surgery with extracorporeal circulation (ECC) can induce microvascular dysfunction and tissue hypoperfusion. We hypothesized that the alterations in near-infrared spectroscopy (NIRS)-derived parameters would be associated with post-operative complications in cardiac surgery patients.

**Methods:**

Prospective observational study performed at two University Hospitals. Ninety patients undergoing cardiac surgery with ECC were enrolled. The NIRS sensor was applied on the thenar eminence. A vascular occlusion test (VOT, 3-min ischemia) was performed at baseline (t0), at Intensive Care Unit (ICU) admission (t1), 3 (t2) and 6 (t3) hours later. Baseline tissue oxygen saturation (StO_2_), oxygen extraction rate and microvascular reactivity indices were calculated.

**Results:**

In the first hours after cardiac surgery, StO_2_ tended to increase (86% [80–89] at T3 versus 82% [79–86] at T0, *p* = ns), while both tissue oxygen extraction and microvascular reactivity tended to decrease, as indicated by increasing occlusion slope (− 8.1%/min [− 11.2 to − 7] at T3 versus − 11.2%/min [− 13.9 to − 7.9] at T0, *p* = ns) and decreasing recovery slope (1.9%/sec [1.1–2.9] at T3 versus 3.1%/sec [2.3–3.9] at T0, *p* = ns). No substantial differences were found in NIRS-derived variables and their changes over time between patients with complications and those without complications.

**Conclusions:**

Peripheral tissue oxygen extraction and microvascular reactivity were reduced during the first hours after cardiac surgery. NIRS-derived parameters were not able to predict complications in this population of cardiac surgery patients.

## Background

Extracorporeal Circulation (ECC) is associated with significant changes in the physiology of peripheral perfusion [1, 2]. The main mechanisms by which ECC leads to an impairment in organ perfusion are the activation of inflammatory pathways with consequent endothelial damage, capillary leak [3], interstitial oedema, and impaired microcirculatory blood flow. Consequently, even when global O_2_ delivery is preserved, a local hypoxia occurs, leading to organ dysfunction that is associated with worse patient outcomes [4]. Near infrared spectroscopy (NIRS) is a non-invasive technique that allows static and dynamic assessments of tissue oxygen saturation in response to ischemic challenge (vascular occlusion test, VOT), thus providing information of peripheral (skeletal muscle) O_2_ extraction rate and microvascular reactivity [5, 6]. Alterations in tissue oxygen saturation are associated with higher mortality in patients with sepsis [7]. NIRS monitoring can provide information on the effects that therapies may have on tissue perfusion and microcirculation [8]. Only a few single-centre studies investigated the relationship between peripheral NIRS-derived parameters and patients’ outcome in cardiac surgery, showing conflicting results and using different devices to assess tissue oxygen saturation [9–15]. Postoperative complications and persistent elevated arterial lactate concentrations were associated with low StO_2_ after ICU admission [9, 12]. By using a vascular occlusion test (VOT), some studies showed that alterations in the desaturation and reperfusion slopes in the early post-operative phase following cardiac surgery were associated with poor outcome, duration of mechanical ventilation, length of ICU stay, and mortality [11, 13, 15]. Conversely, other studies failed to demonstrate the association between NIRS-derived parameters and outcome [10, 14].

The aim of the present bicentric study was to assess the ability of NIRS-derived parameters to predict postoperative complications in patients undergoing cardiac surgery with ECC.

## Methods

This is a prospective observational study carried out at the University Hospitals of Siena and Ancona, Italy. The local ethics committees of both centres approved the study protocol (Local Ethical Committee of University Hospital of Siena-CEL AOUS, date of approval 23/11/2012, and Ethical Committee of Regione Marche-CERM, date of approval 08/11/2013). The patient recruitment started on December 2012 at the University Hospital of Siena and on December 2013 at the University Hospital of Ancona and ended on February 2014 in both centers. Convenience sampling was performed based on the availability of the investigators and/or NIRS device. Written informed consent was obtained from all the included patients the day before surgery. Due to its observational nature, the study was not registered to a specific register. A STrengthening the Reporting of OBservational studies (STROBE) checklist is reported in Additional file 1.

### Study protocol

Adult patients undergoing elective cardiac surgery with ECC were eligible for enrolment in the study. Exclusion criteria were: age < 18 years, surgery without aortic cross-clamping and ECC, surgery with extreme hypothermia or selective brain perfusion, patients undergoing emergency surgery (such as surgery for aortic dissection), and absence of informed consent. The day before surgery (the evening before or the same morning - T0), on admission to the Intensive Care Unit (ICU) (T1), and 3 h (T2) and 6 h (T3) after ICU admission, the patients underwent NIRS monitoring and VOT, as described in detail below. No change of therapy occurred between T0 and the start of surgery. General haemodynamic, blood gas and laboratory parameters were recorded simultaneously. Any type of post-operative major complication (i.e. cardiac, respiratory, renal, abdominal, neurological, haematological, infectious) occurring during stay in the ICU was also recorded. Major complications, including cardiovascular, respiratory, neurological, renal, infectious, haemorrhagic [16], haematological [17], and abdominal [18] events, were defined accordingly to standard definitions (see details in Additional file 2).

### NIRS monitoring

NIRS is a real-time technology that can measure tissue O_2_ saturation (StO_2_) continuously using light in the infrared band to detect differences of absorbance between oxy- and deoxy-haemoglobin [19]. The maximum depth of the tissue sample is estimated to equal half the distance between the probe’s sending and receiving fibers (probe spacing). A light-scattering calibrator is used to normalize the tissue spectrometer during startup of the system and before each measurement. Sample measurement signals are updated every 3.5 s [19]. In this study, an InSpectra StO_2_ Tissue Oxygenation Monitor (model 650; Hutchinson Technology, Hutchinson, MN, USA) was used to measure StO_2_ at baseline and during a VOT with a 15-mm-spaced probe applied on the thenar eminence. The VOT was performed using a pre-defined occlusion time of 3 min. The probe was placed on the hand without the arterial catheter. After a minimum initial 3-min stabilization period (aimed to achieve StO_2_ excursions within ±3% over at least 30 s), arterial blood flow was obstructed by inflating a sphygmomanometer cuff to a pressure of 50 mmHg above systolic arterial pressure. After 3 min of occlusion the cuff was rapidly deflated. StO_2_ was measured continuously until a new stable value was reached. The following parameters were calculated using Inspectra software (InSpectra Analysis Program version 4.00; Hutchinson Technology), as described elsewhere [11, 20] (see Additional file 3 for details):

- baseline StO_2_;
- occlusion slope, an index of tissue O_2_ extraction rate (%/min), calculated from the regression line of StO_2_ decay during the 3 min of blood flow occlusion;
- area of ischemia calculated as area under the occlusion slope curve;
- minimum StO_2_ obtained at the end of the 3-min ischemia;
- recovery slope calculated from the regression line of StO_2_ increase during the reperfusion phase of VOT;
- recovery area or area under the curve of the recovery slope;
- maximum StO_2_ reached during reperfusion;
- area of hyperaemia or the area under the StO_2_ curve reflecting, the phase of reactive hyperaemia following VOT.

### Standard hemodynamic monitoring

After admission to the ICU, central venous pressure (CVP) and arterial pressure were evaluated continuously by an invasive intravascular approach. In patients under mechanical ventilation, cardiac output was obtained using the modified carbon dioxide Fick method (mCO_2_F). Briefly, The mCO_2_F method is based on CO_2_ generation, in which CO_2_ generation and O_2_ consumption are always in a linear relationship [21]. Cardiac output obtained using the mCO_2_F method have showed high accuracy and good reproducibility, to respect classic thermodilution technique [22]. We used the standard formula: CO = VCO_2_/R*da-vO_2_, where VCO_2_ is the carbon dioxide production (ml/min) provided by capnometry, R the Respiratory Exchange Rate, and da-vO_2_ the arterial-venous oxygen difference. An assumed value of the Respiratory Exchange Ratio equal to 0.9 was used for all patients [22].. The cardiac index (CI) was obtained by dividing cardiac output by body surface area. Systemic vascular resistance index (SVRI), oxygen delivery index (DO_2_I) and oxygen extraction ratio (O_2_ER) were calculated with standard formulas. Arterial and venous gas analyses were performed at T0, T1, T2 and T3.

### Anaesthesia, cardiopulmonary bypass, and myocardial protection

Anaesthesia was induced with 1 μg*kg^− 1^ fentanyl, 0.2 mg*kg^− 1^ midazolam or 2 mg*kg^− 1^ propofol, and 0.8 mg*kg^− 1^ rocuronium; 2–2.5% sevoflurane and continuous IV infusion of remifentanil (0.1–0.5 μg*kg^− 1^*min) were used for maintenance. At the beginning of cardio-pulmonary bypass (CPB), sevoflurane was discontinued and replaced with propofol at a maximum infusion rate of 4 mg*kg^− 1^*h IV and a bolus of 5 mg*kg^− 1^ thiopental IV was administered.

ECC was instituted with a roller pump (Terumo Perfusion System 1, Terumo Corporation, Tokyo, Japan), or a centrifugal pump (Revolution Sorin, MedicalExpo, London, UK) and a hollow-fibre oxygenator (Capiox RX 25; Terumo Corporation, Tokyo, Japan) primed with 1200–1500 mL buffered crystalloid solution. During ECC, haemoglobin levels were maintained between 8 and 9 g*dl^− 1^ (haematocrit about 24%). Similar protocols for the ECC were used in the two centers. Myocardial protection was obtained with St. Thomas blood cardioplegia solution or Custodiol solution [23]. The pump flow rate was calculated based on the patient BSA, as follows: Flow (L*min^− 1^*m2) = BSA*2.4. Moderate systemic hypothermia (32–34 °C) was maintained using a heat exchanger connected with ECC machine and α-stat acid-base management was applied.

The patients were weaned off ECC when rectal temperature had reached 34 °C. In the ICU, patients were weaned off mechanical ventilation as soon as they were awake and breathing faster than the ventilator set rate and when the following criteria were met: patient obeying commands; stable and adequate haemodynamics; no significant arrhythmia; core temperature of higher than 36 °C; chest tube drainage of less than 100 mL*h^− 1^ for 2 consecutive hours; diuresis of more than 1 mL*kg^− 1^ per hour; arterial carbon dioxide pressure (PaCO_2_) of less than 50 mmHg; arterial O_2_ pressure (PaO_2_) of more than 70 mmHg; and O_2_ saturation as measured by pulse oxymetry of 92% or higher with the patient breathing less than 50% O_2_.

### Statistical analysis

Statistical analysis was performed using statistical software (SPSS 21.0, SPSS, Chicago, Illinois, and Prism 6.0, GraphPad Software, San Diego, California). The Shapiro-Wilk test was used to test normality of distribution. Data was presented as median [interquartile range], number or percentage, as appropriate. The chi square test was used for comparison of categorical variables. Since the data were not normally distributed, the Mann Whitney U test or the Friedman test with Dunn’s post hoc test for multiple comparisons were used to evaluate differences between groups or over time, as appropriate. Delta values of NIRS-derived variables were also calculated and a two-way analysis of variance (2-way ANOVA) was used to evaluate differences between the two groups at different time points. Binary logistic regression analyses were performed to test the association of changes in NIRS-derived variables and post-operative complications by adjusting for relevant clinical parameters (we constructed one model for each NIRS-derived parameter). A Spearman’s rho was calculated to assess correlations between variables. In order to address the multiple testing problem, the Bonferroni correction was applied to adjust the alpha level of significance. The corrected level of significance after Bonferroni correction for multiple testing was arbitrarily set at α = 0.001. Therefore, an unadjusted *p* value < 0.001 was considered to show statistical significance (all reported *p* values are unadjusted).

## Results

**Fig. 1:**
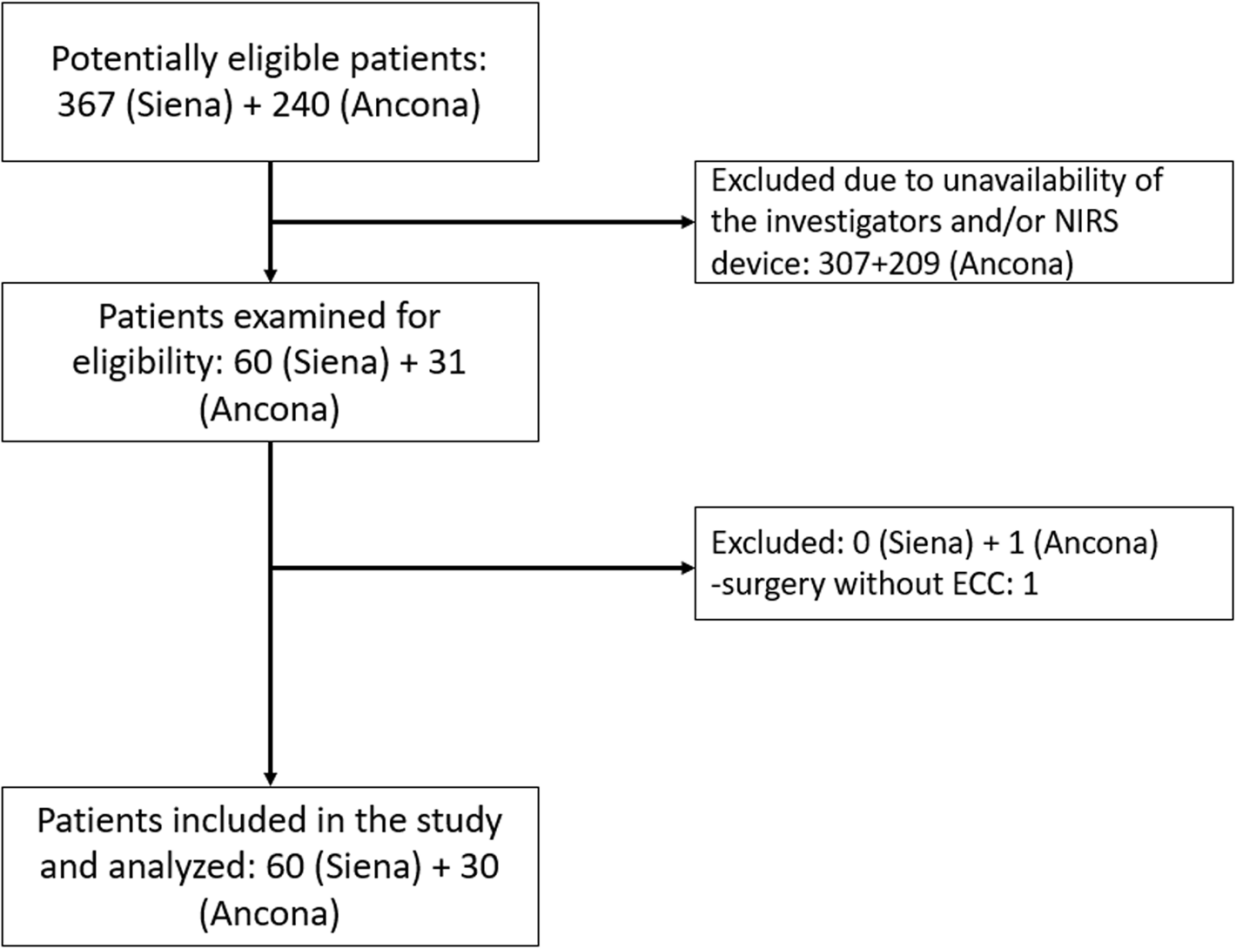
Study flow chart

A convenience sample of 90 patients was studied. Sixty patients were enrolled at the University Hospital of Siena and 30 at the University Hospital of Ancona (Fig. 1). Mean age was 70 [64–76] years and 59 patients (65.6%) were male. The type of surgery was: aortic/mitral valve replacement in 52 patients (58%), coronary artery bypass graft in 17 patients (19%), combined procedures in 17 cases (19%) and other procedures in 4 cases (4%). The logistic Euroscore of the patients was 8.2 ± 7.6, and the length of stay in the ICU was 2 [1–5] days. Thirty-nine patients (43%) developed at least one post-operative complication during their ICU stay. Cardiovascular complications occurred in twenty-seven (30%) patients, respiratory in 13 (14%), renal in 9 (10%), abdominal in 3 (3%), neurological in 6 (7%), haematological (including haemorrhagic) in 3 (3%), and infectious in 4 (4%) patients. Patients with and without complications did not significantly differ in age, gender, presence of comorbidities, duration of ECC or clamping, worst values of mean arterial pressure (MAP), haematocrit, haemoglobin (Hb), SvO_2_, lactate or blood glucose during ECC (Table 1). Variations in hemodynamic parameters are reported in Table 2: no significant differences were found between patients with complications and those without complications.

**Table 1:** General data and intraoperative parameters in patients with or without post-operative complications

	No Complications (*n* = 51)	Complications (*n* = 39)	p
Age (years)	72 [63–76]	70 [65–75]	0.654
Gender (n [% of males])	30 [59%]	29 [74%]	0.174
Comorbidities (n [%])			
*Arterial hypertension*	42 [82%]	27 [69%]	0.208
*Dyslipidemia*	19 [37%]	12 [31%]	0.655
*Coronary Artery Disease*	9 [18%]	12 [31%]	0.208
*Diabetes mellitus*	13 [25%]	6 [15%]	0.303
*Chronic cardiac failure*	9 [18%]	3 [8%]	0.219
*Chronic renal failure*	4 [8%]	8 [20%]	0.117
*Chronic Obstructive Pulmonary Disease*	5 [10%]	4 [10%]	0.999
Duration of ECC (min)	126 [96–167]	116 [81–166]	0.523
Duration of clamping (min)	100 [68–126]	95 [60–129]	0.631
Worst parameters during ECC			
*Min MAP (mmHg)*	60 [50–65]	55 [50–61]	0.158
*Min haematocrit (%)*	25 [22–28]	24 [21–27]	0.771
*Min haemoglobin (g/dL)*	8.0 [7.0–8.8]	7.9 [6.8–9.0]	0.924
*Min SvO*_*2*_ *(%)*	82 [80–86]	81 [78–84]	0.227
*Max lactate (mmol/L)*	2.2 [1.5–3.1]	2.4 [1.9–3.3]	0.337
*Max blood glucose (mg/dL)*	168 [136–186]	156 [137–192]	0.918

Data are expressed as median [1st-3rd quartiles] or numbers and percentages

*ECC* extracorporeal circulation, *MAP* mean arterial pressure, *SvO*_*2*_ venous oxygen saturation

**Table 2:** Variations in haemodynamic parameters in patients with (*n* = 39) or without (*n* = 51) post-operative complications

	t0	t1	t2	t3
MAP (mmHg)				
*All*	91 [83–102]	79 [72–86]*	80 [73–91]*	82 [73–92]*
*No complications*	93 [82–102]	83 [73–90]	84 [77–95]	84 [74–92]
*Complications*	90 [87–102]	77 [68–85]*	75 [71–87]*	78 [72–91]
Heart Rate (bpm)				
*All*	68 [60–75]	85 [79–91]*	87 [80–90]*	86 [80–90]*
*No complications*	68 [57–77]	86 [80–90]*	86 [80–90]*	86 [79–90]*
*Complications*	70 [61–74]	86 [80–93]*	87 [79–90]*	87 [80–90]*
CVP (mmHg)				
*All*	NA	10 [7–12]	9 [7–12]	8 [7–11]
*No complications*	NA	9 [7–12]	8 [7–12]	8 [7–11]
*Complications*	NA	10 [8–13]	10 [8–12]	9 [7–12]
Hb (g/dL)				
*All*	14.1 [13.2–15.3]	11.5 [10.3–12.9]*	12.5 [11.5–13.6]*	12.4 [11.2–13.5]*
*No complications*	14.1 [13.2–15.6]	12 [10.5–13.1]*	12.6 [11.6–13.5]	12.5 [11.1–13.6]*
*Complications*	14.4 [13.2–15]	11.1 [10.2–11.8]*	11.7 [10.6–13.2]	12 [10.4–12.5]
Lactate (mmol/l)				
*All*	1.4 [1.1–1.9]	2.3 [1.5–3.3]*	2.0 [1.4–2.9]*	1.9 [1.6–2.8]*
*No complications*	1.5 [1.2–2]	2.0 [1.3–2.6]	1.8 [1.3–2.7]	1.9 [1.4–2.6]*
*Complications*	1.3 [1.0–1.6]	2.0 [1.4–4.1]	1.8 [1.4–3.5]	1.9 [1.4–3.2]
SvO_2_ (%)				
*All*	NA	72 [66–77]	70 [63–75]	71 [64–76]
*No complications*	NA	71 [67–75]	71 [65–75]	70 [63–76]
*Complications*	NA	74 [63–81]	69 [60–76]	72 [65–81]
CI (L/min/m^2^) (n)				
*All*	NA	2.1 [1.7–2.7] (90)	1.9 [1.5–2.3] (87)	2.2 [1.9–2.5] (66)
*No complications*	NA	2.1 [1.8–2.4] (51)	1.9 [1.6–2.4] (49)	2.1 [1.8–2.5] (34)
*Complications*	NA	2.5 [1.6–3.3] (39)	2.0 [1.5–2.6] (38)	2.2 [1.9–3.1] (32)
SVRI (dyn*s/cm^5^*m^2^)				
*All*	NA	2625 [2250–3415] (90)	3266 [2302–3903] (87)	2639 [2044–3193] (66)
*No complications*	NA	2732 [2195–3284] (51)	3274 [2426–3832] (49)	2747 [2077–3202] (34)
*Complications*	NA	2364 [1958–3090] (39)	2939 [2282–3813] (38)	2643 [1772–3197] (32)
DO_2_I (ml/min/m^2^)				
*All*	NA	337 [231–382] (90)	308 [233–378] (87)	347 [282–425] (66)
*No complications*	NA	337 [257–377] (51)	335 [273–381] (49)	343 [288–409] (34)
*Complications*	NA	341 [218–443] (39)	306 [241–393] (38)	344 [281–436] (32)
O_2_ER (%)				
*All*	NA	26 [22–36] (90)	30 [26–37] (87)	29 [25–35] (66)
*No complications*	NA	26 [23–31] (51)	28 [26–33] (49)	28 [25–35] (34)
*Complications*	NA	27 [22–38] (39)	35 [27–42] (38)	31 [25–35] (32)
Temperature (°C)				
*All*	NA	35.2 [35–36]	36.0 [35.5–36.8]*	36.5 [36.0–37.1]*
*No complications*	NA	35.2 [35–36]	36.0 [35.5–36.9]*	36.5 [36–37]*
*Complications*	NA	35.5 [35–36]	36.2 [35.7–36.8]*	36.7 [36.2–37.3]*
Mechanical ventilation (n [%])				
*All*	–	90 [100%]	87 [97%]	66 [73%]
*No complications*	–	51 [100%]	49 [96%]	34 [67%]
*Complications*	–	39 [100%]	38 [97%]	32 [82%]
Vasopressors (n, %)				
*All*	–	34 [38%]	37 [41%]	33 [37%]
*No complications*	–	16 [31%]	16 [31%]	15 [29%]
*Complications*	–	18 [46%]	21 [54%]	18 [46%]
Inotropic drugs (n, %)				
*All*	–	24 [27%]	25 [28%]	26 [29%]
*No complications*	–	12 [24%]	12 [24%]	11 [22%]
*Complications*	–	12 [30%]	13 [32%]	15 [37%]

Data are expressed as median [1st-3rd quartiles] or numbers and percentages

*p < 0.001 versus baseline, a Friedman test with Dunn’s post hoc test for multiple comparisons was used to evaluate changes over time in each group (according to the Bonferroni correction, an unadjusted *p* < 0.001 was used to indicate statistical significance)

*t0* baseline, *t1* post surgery, *t2* three hours after intensive care unit admission, *t3* six hours after intensive care unit admission, *MAP* mean arterial pressure, *CVP* central venous pressure, *Hb* haemoglobin, *SvO*_*2*_ venous oxygen saturation, *CI* cardiac index, *SVRI* systemic vascular resistance index, *DO*_*2*_*I* oxygen delivery index, *O*_*2*_*ER* oxygen extraction rate

### NIRS-derived parameters and outcome

**Fig. 2:**
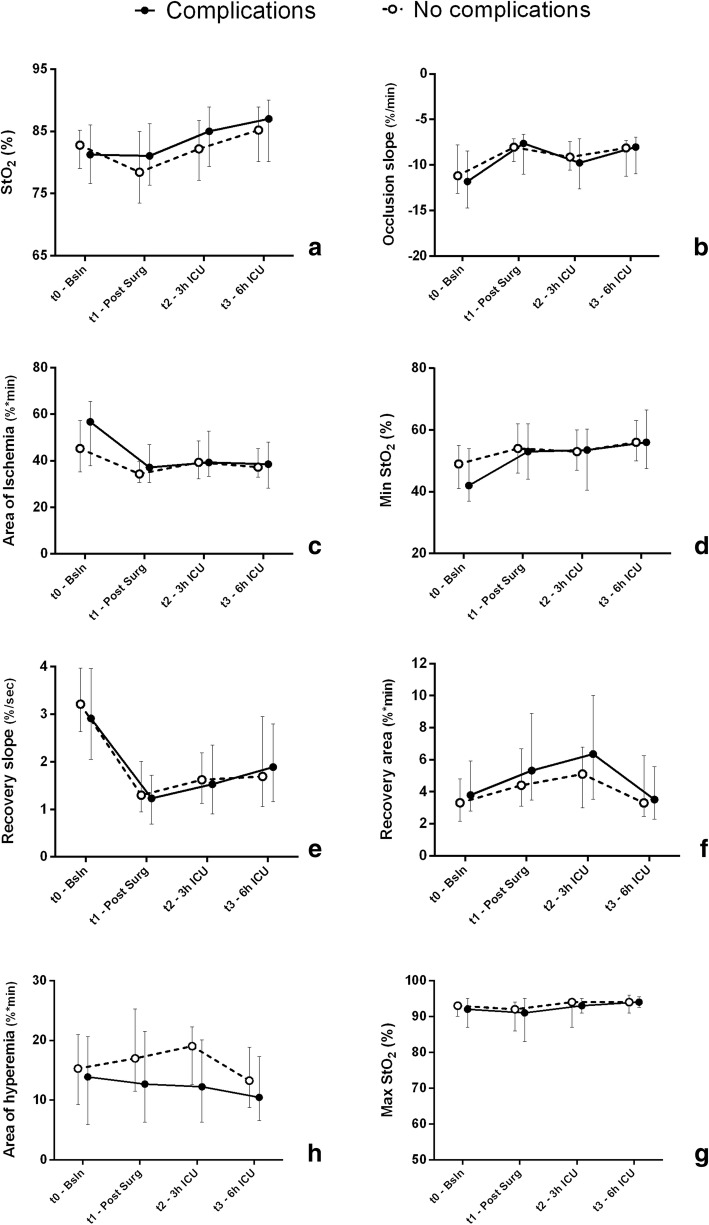
Variations in NIRS-derived variables in patients with or without complications. Data are expressed as median [1st-3rd quartile]. **a** StO_2_,** b** Occlusion slope, **c** Area of ischemia, **d** Min StO_2_, **e** Recovery slope,** f** Recovery area, **g** Max StO_2_, **h** Area of hyperemia

In the overall population, StO_2_ tended to increase above baseline values (86% [80–89] at T3 versus 82% [79–86] at T0, *p* = 0.003). Muscle tissue O_2_ extraction rate tended to be reduced in the first 6 h after surgery, as indicated by a flatter occlusion slope (− 8.1%/min [− 11.2 to − 7] at T3 versus − 11.2%/min [− 13.9 to − 7.9] at T0, *p* = 0.003), smaller area of ischemia (37.4 [31.8–46.5] at T3 versus 47.2 [36.5–60.6] at T0, *p* = 0.002) and higher minimum StO_2_ after 3-min flow occlusion (56% [48–65] at T3 versus 47% [39–55] at T0, *p* < 0.001). Recovery slope was reduced in the first 6 h in ICU (1.9%/sec [1.1–2.9] at T3 versus 3.1%/sec [2.3–3.9] at T0, *p* = 0.001), while recovery area, area of hyperaemia and maximum StO_2_ during recovery were similar to baseline values at T3. Comparison of patients with and without post-operative complications is shown in Fig. 2. No significant differences were found in the variations of NIRS-derived parameters between the two groups (Fig. 2, please see Additional file 4 for comparisons of delta values). Patients with a worsening of NIRS-derived parameters at T3 did not show higher incidence of complications (Additional file 5). Logistic regression analyses (adjusting for age, lactate, MAP, Hb and HR) showed no association of delta values of StO2 (odds ratio 1.068 [95% confidence interval 0.994–1.147], *p* = 0.073), occlusion slope (1.062 [0.963–1.170], *p* = 0.226), recovery slope (1.237 [0.929–1.646], *p* = 0.146) or area of hyperaemia (1.016 [0.962–1.072], *p* = 0.575) at T3 and post-operative complications.

Similar results were obtained comparing patients with cardiovascular complications (*n* = 26, 29%), versus those with other complications (*n* = 13, 14%) versus those without complications (*n* = 51, 57%) (Additional file 6). Correlation analyses between NIRS-derived parameters at ICU systemic admission and intraoperative parameters are reported in Additional file 7. Correlations between NIRS-derived parameters and hemodynamic parameters in pooled data are reported in Additional file 8.

## Discussion

In the present study of 90 patients undergoing cardiac surgery with ECC, NIRS monitoring at the thenar eminence with VOT detected a significant reduction in skeletal muscle microvascular reactivity following surgery, together with a decrease in tissue O_2_ extraction rate, which did not recover in the first 6 h after the operation. We failed to detect an association between NIRS-derived parameters and patient outcome since patients with post-operative complications showed similar variations as those without complications.

It is well known that cardiac surgery with ECC can induce a complex inflammatory response. This can be due to multiple factors, including surgical trauma, haemodilution, ischemia/reperfusion injury, hypothermia and exposure of blood to non-physiological surfaces [1, 3]. The mechanisms involve the following: the release of cytokines, complement activation, leukocyte activation with endothelial adhesion, an increased production of O_2_ free radicals, the release of inflammatory mediators including endothelin, and the deregulation of the nitric oxide pathway [24]. Under these conditions, systemic haemodynamic parameters and markers of global oxygenation, such as central venous O_2_ saturation or arterial lactate, may not be early predictors of tissue hypoperfusion. Although increased lactate levels are related to morbidity and mortality in different patient groups [25], they lack sensitivity and specificity in representing tissue perfusion and may not be sufficient to detect the early impairment of tissue oxygenation [26–28]. The pathological mechanism triggered by the ECC showed strong similarities to those seen during sepsis, potentially leading to impaired microcirculatory perfusion and tissue hypoxia. Studies using sublingual videomicroscopy have shown alterations in microvascular perfusion, which may persist for 24 h after surgery [29] and may occur irrespective of changes in systemic haemodynamics [30]. Bauer et al. showed an increased number of rolling leukocytes in the sublingual microcirculation during CPB, which persisted 1 h after the termination of CPB [31].

NIRS monitoring in conjunction with VOT enables us to estimate peripheral tissue oxygen saturation and microvascular reactivity by evaluating variations in StO_2_ during a brief ischemia/reperfusion test [5]. In several studies using this technology, patients undergoing cardiac surgery showed impaired microvascular reactivity, although conflicting data exists regarding the time needed for recovery to the baseline microvascular state and the relationship with outcome. Smith et al. showed that during CPB, the reperfusion slope decreased as a function of CPB duration, returning to baseline values in all patients within 1 h of the termination of CPB [32]. In another study by Morel et al., StO_2_ and reperfusion slope both declined after CPB but recovered to baseline values after 12 h [10]. Furthermore, these transient changes were not correlated with the patients’ outcome. We found a similar trend in the recovery slope at the first 6 h of the study; however, we did not collect NIRS parameters after 6 h. There are two main differences between our study and that by Morel et al. [10]. Firstly, we used major complications as variables of outcome, instead of using the Sequential Organ Failure Assessment score. Secondly, the duration of VOT was different in the two studies: we applied a three minute time targeted VOT, while Morel et al. maintained the VOT until the StO_2_ value reached 40%. However, despite these differences, our results were consistent with those showed by Morel [10]. Kim et al. demonstrated that the reperfusion slope largely recovered on the first day after surgery in patients without complications, while it remained altered in those with complications [12]. In the present study, the recovery slope remained reduced in patients for 6 h after admission to the ICU, indicating a persistent decrease in microvascular reactivity. However, we were unable to detect more severe alterations or delayed recovery of this parameter among patients with post-operative complications in such a short monitoring period. There may be several explanations for these discrepant results. First, we performed 3-min blood flow occlusion in all patients, instead of using a target StO_2_, and this may have produced different degrees of ischemia. It has been shown that StO_2_ recovery rate depends on the minimum StO_2_ reached after 3 min of blood flow occlusion; i.e. the velocity of reperfusion and the degree of hyperaemia are related to the degree of ischemia [33]. Second, it is possible that the monitoring period in this study was too short to detect differences for predicting post-operative morbidity.

Kopp et al. showed that StO_2_ was reduced after cardiac surgery and that the minimum value of StO_2_ in the first hours after the operation was predictive of delayed lactate clearance [13]. However, in a similar patient population other authors have found a transient increase in StO_2_ after surgery [34]. StO_2_ reflects the balance between regional O_2_ delivery and consumption [5]. In our study, in the first 6 h after surgery, we observed an increase in StO_2_, a slower desaturation rate (flatter occlusion slope), smaller area of ischemia, and increased StO_2_ values (nadir) during 3-min of ischemia, but no difference was seen between patients with and without complications. Taken together, these findings suggest a reduction in regional O_2_ consumption, despite systemic O_2_ER resulting in the normal range in both groups of patients. Several factors may influence O_2_ extraction and consumption in skeletal muscle, including the administration of sedative or vasopressor agents, a residual neuromuscular blockade in the first post-operative hours, and variations in body temperature [4, 35].

Our study has several limitations. First of all, we studied a convenience sample of 90 patients without preliminary statistical calculation of the required sample size. Based on previous studies, we retrospectively calculated that the inclusion of a total of 96 patients was required to show a difference in the reperfusion slope between patients with, and those without, post-operative complications (calculated Cohen’s d = 0.58 [12]) with a power of 80% and an alpha error of 0.05. Therefore, our study may be slightly underpowered, although it is highly unlikely that the inclusion of 6 additional patients would have changed our results substantially. Second, by applying NIRS to the thenar eminence, we evaluated tissue oxygen saturation and microvascular reactivity in a peripheral tissue. We cannot determine whether similar alterations were induced in microvascular beds of the splanchnic organs. We used the thenar eminence because the thickness of the adipose tissue covering this muscle is small. Although tissue oedema can increase the thickness of the subcutaneous layer, this hardly happens in a period of time as short as that of our study [20]. Third, since the degree of neuromuscular blockade was not measured in our patients, it is possible that the reduction in muscle O_2_ extraction rate may at least partly depend on a residual neuromuscular blockade in the early postoperative period. Moreover, the use of vasopressors, inotropes and transfusions, as well as the perioperative fluid balance, could affect peripheral tissue oxygen supply and utilization. Unfortunately however, we could not evaluate the potential influence of these treatments because data on vasoactive dosage or fluid and transfusion requirements were not collected. Similarly, we were unable to evaluate the impact of different ECC protocols (e.g the use of roller versus centrifugal pump). Fourth, we did not perform intraoperative NIRS measurements that could provide information about earlier variations in tissue oxygen saturation and microvascular function and their impact on clinical outcomes. Fifth, a target StO_2_ for VOT, instead of a pre-defined occlusion time, may have been more appropriate for standardizing the degree of ischemia and the hyperaemic phase [33]. Furthermore, we did not collect the tissue haemoglobin index, usually used to calculate the muscle oxygen consumption (NirVO_2_); therefore, we could not evaluate the impact of variations of the NirVO_2_ on the outcome. Sixth, we did not evaluate the potential role of intraoperative complications or risky events (e.g., arterial hypotension, haemorrhage, low cardiac output) on NIRS parameter and their relationship with the outcome. Again, the tissue spectrometer (InSpectra Model 650) used in the present study, and the company that produces it (Hutchinson Technology, Hutchinson, MN, USA) are actually out of the market. Similar devices using near infrared spectroscopy exist that can provide tissue O_2_ saturation. Nonetheless, it must be recognized that differences in the proprietary algorithms of different NIRS devices make the comparisons between studies difficult.

Finally, we performed multiple comparisons on a number of variables, thus our analysis may be affected by bias due to multiple-testing and data coupling problems. Indeed, the slopes for the decreasing and increasing StO2 during the VOT are not independent from the areas that are defined by the slopes; therefore, a data coupling may occur. Nonetheless, we applied the Bonferroni correction to enhance the robustness of our results, and NIRS variables were included individually in separate logistic regression models in order to avoid the problem of collinearity. Even if the study is observational, not having registered it in any public register, the designs and statistical analyses are unverifiable.

## Conclusions

In patients undergoing cardiac surgery with ECC, thenar NIRS monitoring in conjunction with VOT in the first 6 h after the operation showed a reduction in peripheral tissue O_2_ extraction and microvascular reactivity. NIRS-derived parameters were not able to predict postoperative complications in this population of cardiac surgery patients.

## Supplementary information

**Additional file 1.** STROBE checklist.

**Additional file 2.** Definitions of post-operative complications.

**Additional file 3.** NIRS monitoring technique.

**Additional file 4.** Comparisons of delta values of NIRS-derived variables between patients with complications and those without complications. Data are expressed as mean ± standard deviation. Repeated measures 2-way ANOVA with Bonferroni post hoc test.

**Additional file 5.** Incidence of post-operative complications among patients with a worsening in NIRS-derived variables at T3.

**Additional file 6. **Variations in NIRS derived parameters in patients with post-operative cardiac complications (*n* = 26) versus those with other complications (*n* = 13) versus those without complications (*n* = 51).

**Additional file 7.** Correlation analyses between NIRS-derived parameters at T1 (time of admission to the ICU) and intraoperative parameters.

**Additional file 8.** Correlation analyses between NIRS-derived parameters and hemodynamic parameters in pooled data.

## Data Availability

The datasets generated and analysed during the current study are available from the corresponding author on reasonable request.

## Abbreviations

BSA: Body surface area
CI: Cardiac index
CPB: Cardiopulmonary bypass
CVP: Central venous pressure
DO_2_I: Systemic oxygen delivery index
ECC: Extracorporeal circulation
Hb: Haemoglobin
ICU: Intensive care unit
MAP: Mean arterial pressure
NIRS: Near infrared spectroscopy
NirVO_2_: Muscle oxygen consumption
O_2_: Oxygen
O_2_ER: Oxygen extraction rati
PaCO_2_: Arterial carbon dioxide pressure
PaO_2_: Arterial oxygen pressure
StO_2_: Tissue oxygen saturation
SvO_2_: Venous oxygen saturation
SVRI: Systemic vascular resistance index
VOT: Vascular occlusion test
