# Supplementary material for: Tissue oxygen saturation changes and postoperative complications in cardiac surgery: a prospective observational study
Source: BMC Anesthesiol. 2019 Dec 16;19:229. doi: 10.1186/s12871-019-0905-5 (PMC6916088; doi:10.1186/s12871-019-0905-5)
Supplement: Supplementary file 2 — Additional file 2. Definitions of post-operative complications. [file 12871_2019_905_MOESM2_ESM.docx]

**Definitions of post-operative complications**

1) cardiovascular complications: myocardial infarction (documented by electrocardiography and enzyme criteria); low cardiac output requiring inotropic support for >24 hours, an intra-aortic balloon pump (IABP) or a ventricular assist device; or severe arrhythmias requiring treatment or cardiopulmonary resuscitation.

2) respiratory complications: prolonged ventilatory support (defined as mechanical ventilatory support for >24 hours); re-intubation; tracheostomy; clinical evidence of pulmonary embolism or edema; adult respiratory distress syndrome.

3) neurological complications: central nervous system complications (defined as a focal brain lesion (confirmed by clinical findings or computed tomographic scan, or both), diffuse encephalopathy with >24 hours of severely altered mental status or unexplained failure to awaken within 24 hours of the operation.

4) renal complications: acute renal failure (need for dialysis).

5) infectious complications: culture-proven pneumonia, mediastinitis, wound infection, septicemia (with appropriate clinical findings) or septic shock.

6) abdominal complications: gastrointestinal haemorrhage, perforated ulcer, pancreatitis/hyperamylasaemia, acute cholecystitis, bowel ischaemia/ischaemic colitis, diverticulitis, liver dysfunction

7) hematologic complications: bleeding requiring re-operation; heparin resistance (HR) (failure to reach an ACT longer than 480 s after an intravenous bolus administration of 300 IU/kg heparin and/or a heparin sensitivity index below 1.0. AT III-independent HR was defined as HR in a patient with a plasmatic level of AT III ≥100%.
