# Supplementary material for: Tissue oxygen saturation changes and postoperative complications in cardiac surgery: a prospective observational study
Source: BMC Anesthesiol. 2019 Dec 16;19:229. doi: 10.1186/s12871-019-0905-5 (PMC6916088; doi:10.1186/s12871-019-0905-5)
Supplement: Supplementary file 3 — Additional file 3. NIRS monitoring technique. [file 12871_2019_905_MOESM3_ESM.docx]

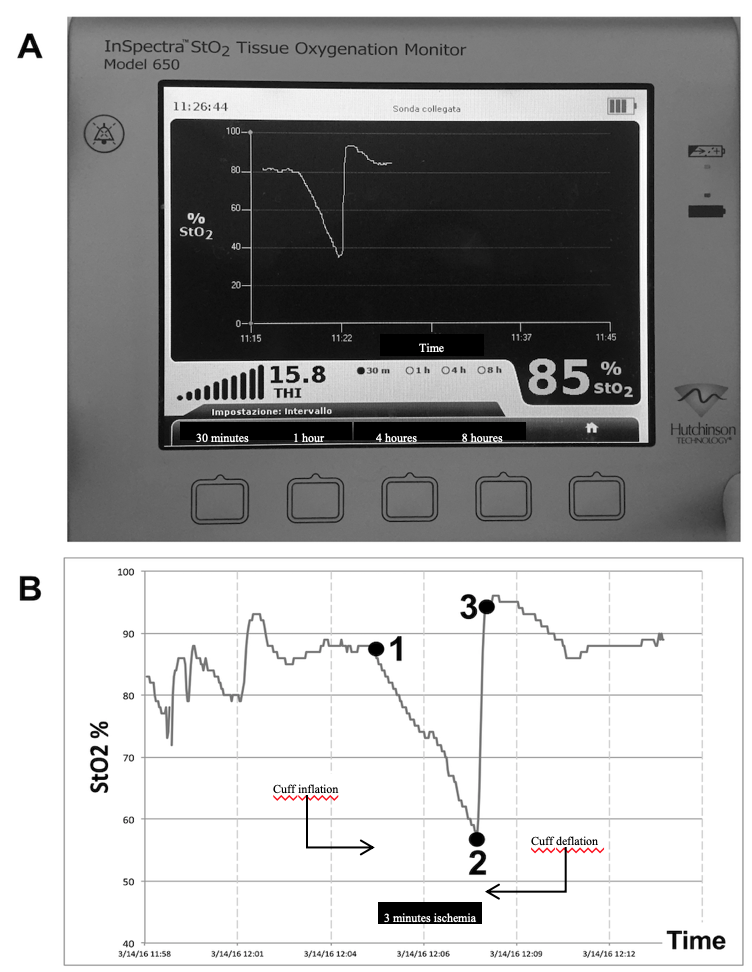


A. An example of the near-infrared spectrophotometer monitor (Inspectra, Hutchinson Technology Incorporated). The monitor shows the tissue oxygen saturation (StO_2_) trend over time during a vascular occlusion test (VOT, see text for details). Right bottom, the instantaneous value of StO_2_. Left bottom, the instantaneous value of tissue hemoglobin index (THI), which is a hemoglobin signal strength metric.

B. An example of vascular occlusion test (VOT). The graph shows the trend of StO_2_ (y-axis) over the time (x-axis). The arterial blood flow was obstructed by inflating a sphygmomanometer cuff to a pressure of 50 mmHg above systolic arterial pressure (1). After 3 minutes of VOT the sphygmomanometer cuff was rapidly deflated (2) and the blood flow increased quickly (3) up to the maximum StO2 value. The first degree slope (from point 1 to point 2 of the figure) of the hemoglobin desaturation curve during ischemia reflects the tissue oxygen consumption rate (%/min), and the slope of the increase (from point 2 to point 3) of StO2 after the release of the brachial vascular occlusion is indicative of the reperfusion rate (%/min). The area under occlusion slope, called area of ischemia, increases with an increased regional oxygen extraction. The recovery area (the area under the curve of the recovery slope) is indicative of improved perfusion and/or microvascular integrity. See the text for a complete list of parameters obtained with the VOT.
