# Supplementary figures and images for: Tissue oxygen saturation changes and postoperative complications in cardiac surgery: a prospective observational study
Source: BMC Anesthesiol. 2019 Dec 16;19:229. doi: 10.1186/s12871-019-0905-5 (PMC6916088; doi:10.1186/s12871-019-0905-5)

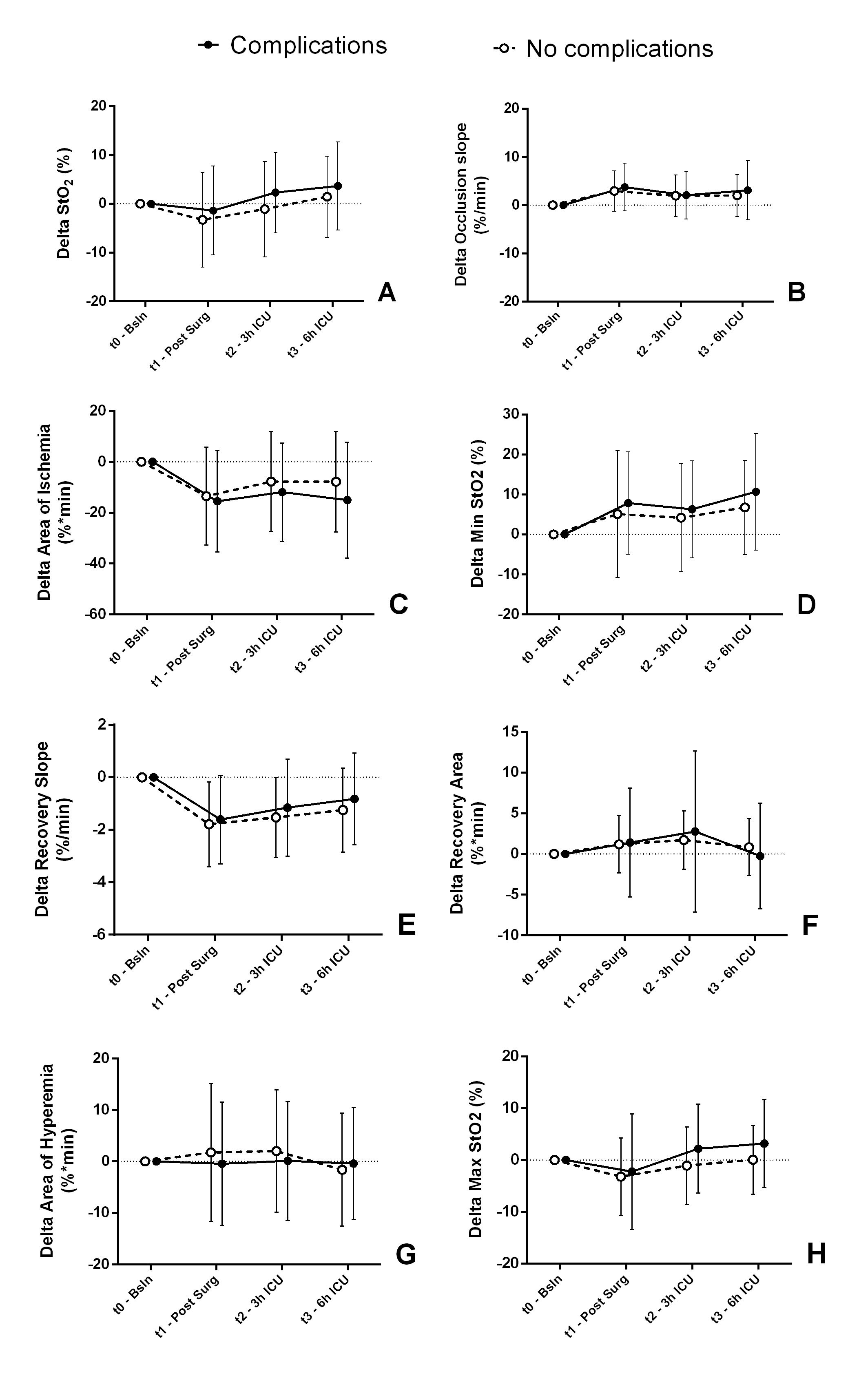

Supplement: Supplementary file 4 — Additional file 4. Comparisons of delta values of NIRS-derived variables between patients with complications and those without complications. Data are expressed as mean ± standard deviation. Repeated measures 2-way ANOVA with Bonferroni post hoc test. [file 12871_2019_905_MOESM4_ESM.jpg]
