# Supplementary material for: Tissue oxygen saturation changes and postoperative complications in cardiac surgery: a prospective observational study
Source: BMC Anesthesiol. 2019 Dec 16;19:229. doi: 10.1186/s12871-019-0905-5 (PMC6916088; doi:10.1186/s12871-019-0905-5)
Supplement: Supplementary file 5 — Additional file 5. Incidence of post-operative complications among patients with a worsening in NIRS-derived variables at T3. [file 12871_2019_905_MOESM5_ESM.docx]

**Additional File 3 – Incidence of post-operative complications among patients with a worsening in NIRS-derived variables at T3.**

|  | All complications (n, %) |  | Cardiac complications (n, %) |  |
| --- | --- | --- | --- | --- |
| StO2 at 6 hours |  |  |  |  |
| *worsening* | 11/29 (38%) | p=0.315 | 8/29 (28%) | p=0.529 |
| *improvement* | 28/61 (46%) |  | 18/61 (29%) |  |
| Occlusion slope at 6 hours |  |  |  |  |
| *worsening* | 22/58 (38%) | p=0.121 | 15/58 (26%) | p=0.269 |
| *improvement* | 17/32 (53%) |  | 11/32 (34%) |  |
| Recovery slope at 6 hours |  |  |  |  |
| *worsening* | 24/64 (37%) | p=0.065 | 13/64 (20%) | p=0.006 |
| *improvement* | 15/26 (58%) |  | 13/26 (50%) |  |
| Area of hyperemia |  |  |  |  |
| *worsening* | 18/45 (40%) | p=0.391 | 10/45 (22%) | p=0.179 |
| *improvement* | 19/42 (45%) |  | 14/42 (33%) |  |

Worsening = any reduction in StO2, recovery slope or area of hyperemia, or any increase in the occlusion slope as compared to baseline values.

Chi-square test with Bonferroni correction, a p<0.001 was considered to indicate statistical significance.
