# Supplementary material for: Tissue oxygen saturation changes and postoperative complications in cardiac surgery: a prospective observational study
Source: BMC Anesthesiol. 2019 Dec 16;19:229. doi: 10.1186/s12871-019-0905-5 (PMC6916088; doi:10.1186/s12871-019-0905-5)
Supplement: Supplementary file 6 — Additional file 6. Variations in NIRS derived parameters in patients with post-operative cardiac complications (n = 26) versus those with other complications (n = 13) versus those without complications (n = 51). [file 12871_2019_905_MOESM6_ESM.docx]

**Additional File 6**

**Variations in NIRS derived parameters in patients with post-operative cardiac complications (n=26) versus those with other complications (n=13) versus those without complications (n=51).**

|  | **t0** | **t1** | **t2** | **t3** |
| --- | --- | --- | --- | --- |
| StO_2_ (%) |  |  |  |  |
| *No complications* | 83 [79-85] | 78 [73-85] | 82 [77-87] | 85 [80-89] |
| *Cardiac Complications* | 82 [77-86] | 82 [77-86] | 85 [77-91] | 88 [82-92] |
| *Other Complications* | 80 [75-86] | 79 [75-86] | 84 [80-87] | 83 [80-88] |
| Occlusion slope (%/min) |  |  |  |  |
| *No complications* | −11.2 [−7.8—13.1] | −8.0 [−7.1-−9.6] # | −9.1 [−7.4-−10.6] | −8.1 [−7.3-−11.2] |
| *Cardiac Complications* | −12.9 [−8.8—15.6] | −7.5 [−6.5-−9.4] | −9.7 [−6.3-−12.3] | −7.8 [−6.9-−11.4] |
| *Other Complications* | −11.5 [−13.2—7.6] | −8.1 [−6.6—12.0] | −9.8 [−7.6—14.7] | −8.0 [−6.7—10.3] |
| Area of Ischemia (%*min) |  |  |  |  |
| *No complications* | 45 [35-77] | 34 [30-39] | 39 [32-49] | 37 [33-45] |
| *Cardiac Complications* | 58 [42-66] | 37 [31-44] # | 40 [35-52] | 37 [26-53] |
| *Other Complications* | 48 [33-64] | 37 [30-49] | 39 [28-55] | 42 [29-45] |
| Min StO_2_ (%) |  |  |  |  |
| *No complications* | 49 [41-55] | 54 [46-62] | 53 [47-60] | 56 [50-63] |
| *Cardiac Complications* | 42 [37-51] | 53 [46-64] | 55 [42-64] | 62 [47-69] # |
| *Other Complications* | 43 [38-61] | 51 [41-57] | 49 [39-57] | 55 [49-59] |
| Recovery slope (%/sec) |  |  |  |  |
| *No complications* | 3.2 [2.6-4.0] | 1.3 [0.9-2.0] # | 1.6 [1.1-2.2] # | 1.7 [1.1-2.9] # |
| *Cardiac Complications* | 2.6 [1.9-3.7] | 1.4 [0.7-1.7] | 1.6 [1.0-2.5] | 1.9 [1.1-2.8] |
| *Other Complications* | 3.4 [2.5-5.3] | 1.1 [0.8-1.5] # | 1.3 [0.8-2.2] | 1.8 [1.5-2.8] |
| Recovery area (%*sec) |  |  |  |  |
| *No complications* | 3.3 [2.2-4.8] | 4.4 [3.1-6.7] | 5.1 [3.0-6.8] | 3.3 [2.5-6.3] |
| *Cardiac Complications* | 3.8 [2.6-5.9] | 4.5 [3.8-7.2] | 5.3 [3.3-8.5] | 3.5 [2.3-5.4] |
| *Other Complications* | 3.1 [2.4-5.9] | 8.0 [3.2-15.2] | 8.1 [5.2-10.6] | 3.6 [2.3-7.5] |
| Area of hyperemia (%*min) |  |  |  |  |
| *No complications* | 15.4 [9.4-21.9] | 17.0 [11.6-25.2] | 18.8 [12.5-22.2] | 12.8 [8.8-18.7] |
| *Cardiac Complications* | 10.0 [3.6-21.6] * | 12.7 [3.6-21.6] | 12.1 [4.5-20.6] | 9.4 [3.7-15.3] |
| *Other Complications* | 19.6 [14.0-26.9] † | 13.1 [6.7-19.8] | 11.3 [6.7-18.5] | 13.9 [8.8-22.1] |
| Max StO_2_ (%) |  |  |  |  |
| *No complications* | 93 [90-94] | 92 [86-94] | 94 [87-95] | 94 [91-96] |
| *Cardiac Complications* | 92 [84-95] | 91 [83-95] | 94 [91-96] | 94 [92-96] |
| *Other Complications* | 94 [89-95] | 90 [82-94] | 92 [91-94] | 94 [93-94] |

StO_2_, oxygen tissue saturation; Min StO_2_, minimum value of oxygen tissue saturation; Max StO_2_, maximum value of oxygen tissue saturation.

#p<0.001 versus baseline (Friedman test with Dunn’s test for multiple comparisons and Bonferroni correction for multiple testing)

*p=0.0279 versus patients without complications, † p=0.0226 versus patients with cardiac complications (Kruskal-Wallis test). Corrected level of significance after Bonferroni correction for multiple testing would be α=0.001, therefore the observed differences are not statistically significant.
