# Supplementary material for: Tissue oxygen saturation changes and postoperative complications in cardiac surgery: a prospective observational study
Source: BMC Anesthesiol. 2019 Dec 16;19:229. doi: 10.1186/s12871-019-0905-5 (PMC6916088; doi:10.1186/s12871-019-0905-5)
Supplement: Supplementary file 7 — Additional file 7. Correlation analyses between NIRS-derived parameters at T1 (time of admission to the ICU) and intraoperative parameters. [file 12871_2019_905_MOESM7_ESM.docx]

**Additional File 7 – Correlation analyses between NIRS-derived parameters at T1 (time of admission to the ICU) and intraoperative parameters.**

|  | Time of ECC (min) | Time of clamping (min) | Max Lactate (mmol/l) | Max glucose (mg/dl) | Lowest MAP (mmHg) | Lowest Hct (%) | Lowest ScvO2 (%) |
| --- | --- | --- | --- | --- | --- | --- | --- |
| StO2 (%) | r=0.27, p=0.011 | r=0.24, p=0.022 | r=0.41, p<0.001 | r=0.41, p<0.001 | r=-0.17, p=0.104 | r=-0.05, p=0.660 | r=-0.17, p=0.123 |
| Occlusion slope (%/min) | r=-0.17, p=0.102 | r=-0.25, p=0.016 | r=-0.34, p=0.001 | r=-0.06, p=0.589 | r=0.10, p=0.329 | r=0.13, p=0.223 | r=-0.12, p=0.265 |
| Occlusion area (%*min) | r=0.09, p=0.394 | r=0.13, p=0.238 | r=0.28, p=0.007 | r=0.20, p=0.063 | r=-0.19, p=0.073 | r=-0.09, p=0.388 | r=0.02, p=0.852 |
| Minimum StO2 (%) | r=0.12, p=0.254 | r=0.06, p=0.591 | r=0.10, p=0.357 | r=0.22, p=0.033 | r=-0.03, p=0.811 | r=0.10, p=0.358 | r=-0.15, p=0.175 |
| Recovery slope (%/min) | r=0.06, p=0.571 | r=0.17, p=0.117 | r=0.14, p=0.179 | r=0.05, p=0.179 | r=-0.07, p=0.500 | r=-0.17, p=0.111 | r=0.13, p=0.220 |
| Recovery area (%*min) | r=0.26, p=0.011 | r=0.23, p=0.027 | r=0.31, p=0.003 | r=0.23, p=0.030 | r=-0.08, p=0.434 | r=0.00, p=0.998 | r=0.05, p=0.637 |
| Maximum StO2 (%) | r=0.20, p=0.064 | r=0.23, p=0.029 | r=0.36, p<0.001 | r=0.33, p=0.001 | r=-0.17, p=0.119 | r=-0.05, p=0.640 | r=-0.02, p=0.877 |
| Area of hyperemia (%*min) | r=-0.20, p=0.063 | r=-0.11, p=0.293 | r=-0.14, p=0.181 | r=-0.15, p=0.146 | r=0.10, p=0.348 | r=0.02, p=0.871 | r=0.18, p=0.084 |

Spearman correlation, with Bonferroni correction. A p<0.001 is considered to indicate statistical significance.

*ECC* Extracorporeal Circulation, *MAP* mean arterial pressure, *Hct* Hematocrit, *ScvO2* central venous O2 saturation
