# Supplementary material for: Tissue oxygen saturation changes and postoperative complications in cardiac surgery: a prospective observational study
Source: BMC Anesthesiol. 2019 Dec 16;19:229. doi: 10.1186/s12871-019-0905-5 (PMC6916088; doi:10.1186/s12871-019-0905-5)
Supplement: Supplementary file 8 — Additional file 8. Correlation analyses between NIRS-derived parameters and hemodynamic parameters in pooled data. [file 12871_2019_905_MOESM8_ESM.docx]

**Additional File 8 – Correlation analyses between NIRS-derived parameters and hemodynamic parameters in pooled data.**

|  | CI (ml/min/m2) | DO_2_I (ml/min/m2) | ScvO_2_ (%) | O_2_ER (%) | SVRI (dyn*s/cm5*m2) | MAP (mmHg) | Lactate (mmol/l) |
| --- | --- | --- | --- | --- | --- | --- | --- |
| StO2 (%) | r=0.25, p=0.001 | r=0.32, p<0.001 | r=0.28, p<0.001 | r=-0.31, p<0.001 | r=-0.27, p<0.001 | r=-0.07, p=0.205 | r=0.19, p<0.001 |
| Occlusion slope (%/min) | r=0.18, p=0.023 | r=0.17, p=0.035 | r=0.10, p=0.106 | r=-0.13, p=0.078 | r=-0.17, p=0.034 | r=-0.15, p=0.005 | r=0.11, p=0.061 |
| Occlusion area (%*min) | r=-0.13, p=0.101 | r=-0.10, p=0.187 | r=-0.08, p=0.195 | r=0.10, p=0.170 | r=0.08, p=0.301 | r=0.06, p=0.258 | r=-0.14, p=0.016 |
| Minimum StO2 (%) | r=0.24, p=0.002 | r=0.26, p=0.001 | r=0.25, p<0.001 | r=-0.27, p<0.001 | r=-0.22, p=0.004 | r=-0.11, p=0.045 | r=0.20, p<0.001 |
| Recovery slope (%/min) | r=-0.03, p=0.675 | r=-0.03, p=0.683 | r=0.08, p=0.196 | r=-0.09, p=0.259 | r=0.18, p=0.021 | r=0.33, p<0.001 | r=-0.26, p<0.001 |
| Recovery area (%*min) | r=-0.20, p=0.013 | r=-0.20, p=0.017 | r=-0.33, p<0.001 | r=0.29, p<0.001 | r=0.01, p=0.879 | r=-0.19, p<0.001 | r=0.17, p=0.003 |
| Maximum StO2 (%) | r=0.18, p=0.024 | r=0.24, p=0.002 | r=0.17, p=0.006 | r=-0.28, p<0.001 | r=-0.14, p=0.067 | r=0.05, p=0.324 | r=0.12, p=0.040 |
| Area of hyperemia (%*min) | r=-0.18, p=0.025 | r=-0.17, p=0.030 | r=-0.16, p=0.011 | r=0.05, p=0.502 | r=0.31, p<0.001 | r=0.16, p=0.002 | r=-0.10, p=0.082 |

Spearman correlation, with Bonferroni correction. A p<0.001 is considered to indicate statistical significance.

*CI* cardiac index, *DO_2_I* oxygen delivery index, *ScvO_2_* central venous O_2_ saturation, *O_2_ER* oxygen extraction ratio, *SVRI* systemic vascular resistance index, *MAP* mean arterial pressure
